# Robotic-Assisted Percutaneous Coronary Intervention: Rationale, Implementation, Case Selection and Limitations of Current Technology

**DOI:** 10.3390/jcm7020023

**Published:** 2018-01-30

**Authors:** Michael Ragosta, Kanwar P. Singh

**Affiliations:** Cardiovascular Division, University of Virginia Health System, Charlottesville, VA 22908, USA; kps2u@virginia.edu

**Keywords:** coronary intervention, robotics, coronary artery disease, radiation exposure, occupational hazards

## Abstract

Interventional cardiologists have witnessed an explosive growth in the field. A wide array of percutaneous procedures allow us to treat numerous cardiac conditions less invasively. However, the way we work has changed very little over the past decades. We continue to stand at the tableside for prolonged periods of time, exposing ourselves to the very real risks of radiation exposure as well as to the associated orthopedic injuries from radiation protection. The precision of our procedures is limited by the distance from the fluoroscopic images and, furthermore, patients are potentially at risk from operator fatigue caused by a physician standing at the table for prolonged periods while wearing cumbersome radiation protection gear. Robotic-assisted coronary intervention removes the operator from the radiation field and has been shown to markedly reduce operator exposure as well as allow for more precise positioning of balloons and stents. This technology holds great promise for making interventional procedures safer and more comfortable for the operators as well as reducing fatigue, potentially improving patient outcomes. Currently, we are in an ‘early adopter’ phase of this technology and this paper reviews the rationale, methodology, optimal case selection, and limitations of robotic-assisted coronary intervention.

## 1. Introduction

Ever since the first coronary angiogram in 1958, the field of interventional cardiology has witnessed explosive growth due to the major discoveries in vascular biology in concert with the development of an impressive array of drugs, devices, and techniques. Currently, we can treat a broad spectrum of coronary artery, peripheral artery, and structural heart diseases with less invasive, percutaneous approaches that we did not dream possible just a decade ago. It is certain that our interventional repertoire will see further growth and we will be able to treat more conditions by percutaneous methods previously thought impossible.

Despite the impressive array of what we are currently able to do, it is interesting to note that there has been little change in how we do it. A time traveler visiting the catheterization laboratory of Mason Sones performing a coronary angiogram would witness a familiar scene: a patient lying uncomfortably on a procedure table with the physician standing (uncomfortably) at their side, near the X-ray equipment and wearing a heavy, leaded garment while Dr. Sones manipulates a catheter and cranes his neck to look at the X-ray image across the procedure table from where he stands. Although the equipment has become more modern and the tricks we perform more complex, the way we work has not changed in 60 years.

The enormous advances in interventional cardiology have naturally focused on improving a procedure’s safety and efficacy for the patient. There has been minimal-to-no effort invested in improving the safety or comfort of the procedure for the physician. The occupational hazards associated with a career’s worth of radiation exposure as well as those hazards associated with years of prolonged standing while wearing heavy leaded garments to protect oneself from radiation have not been given adequate attention. Further, the effect of fatigue from prolonged standing while wearing leaded garments on the outcome of the procedure has not been studied at all. As a profession, we have not been as careful nor as creative with the development of our own protection regarding the occupational hazards of our field, nor have we addressed the impact of fatigue on procedural outcomes. The nascent field of robotic-assisted coronary intervention is really the first significant advance in our field focusing on these issues.

## 2. Radiation Hazards to the Interventional Cardiologist

Radiation safety is part of fellowship training, yet is often relegated to a one-hour lecture or a mandatory web-based training module. Fellows often develop sloppy radiation safety practices which are rarely corrected by the attending supervisor. As a whole, our profession can do a much better job of implementing radiation safety practices. Nevertheless, even with meticulous attention to sound radiation safety practices, we are still exposed to a significant amount of ionizing radiation exposure during our careers and the risks are very real (Table 1).

Interventional cardiologists have the highest radiation exposure of all health care workers [1,2]. Interventional cardiologists have estimated exposures of about 5 mSv per year, 2–3 times higher than radiologists [3]. This translates to an increased lifetime risk of cancer 1 per 100 exposed individuals [3,4]. Several specific malignancies are associated with occupational radiation exposure. An increased risk of basal cell skin cancers has been observed in X-ray technicians [5]. Hematologic malignancies have been clearly associated with radiation exposure, but most of this data was observed in atomic bomb survivors, who received brief, high dose exposures. The risk of low dose exposure over many years as seen in occupational workers is less clear. One study of over 300,000 radiation workers in nuclear power plants with average exposures of 1.1 mGy per year observed an increased risk of chronic myelogenous leukemia, but not chronic lymphocytic leukemia, lymphoma, or myeloma [6]. Occupational radiation exposure is also associated with thyroid cancer [7]. A recent report associating malignant brain tumors with radiation exposure generated great interest in our community [8]. This observational study found 31 physicians with brain tumors; over half of these consisted of glioblastoma multiforme. Most of the cases (*n* = 23) occurred in interventional cardiologists with an average of 23.5 years of exposure to ionizing radiation. Of particular concern, 85% of the tumors were left-sided, consistent with the greater operator exposure of the left side of the head.

Cancer is not the only risk of radiation. Ionizing radiation increases the risk of cataracts with 50% of interventional cardiologists noted to have posterior subcapsular changes that are not due to age and are typically observed with radiation [9]. Chronic, low level radiation has effects on the reproductive system, including a reduction in sperm count and an increased risk of spontaneous abortion, congenital defects, low birth weight, and teratogenesis when exposed early in pregnancy [10]. An increased risk of left-sided carotid atherosclerosis along with vascular changes associated with aging has been observed in interventional cardiologists [11]. Finally, the interventional cardiologist having trouble remembering where their car is parked may be interested to read a recent report finding impairment in short term memory and verbal fluency in interventional cardiologists compared to controls without radiation exposure [12]. This finding is consistent with the known entity of radiation-induced neurodegenerative changes in the brain seen with therapeutic brain irradiation and, in fact, the total cumulative dose received by an interventional cardiologist over their career may approach the therapeutic doses used in oncology [13].

## 3. Orthopedic Hazards to the Interventional Cardiologist

Prolonged standing while wearing leaded garments clearly takes its toll on the operator. It is doubtful that a practicing interventional cardiologist needs data to convince them of this point, however, there are several published reports describing the magnitude of occupational injury. The Society of Cardiovascular Angiography and Interventions surveyed its membership on the frequency and location of work-related injury and reported their findings in 2004 and again in 2015 [14,15]. The respondents were high-volume and experienced operators. Orthopedic injuries were very common, with roughly half suffering from at least one orthopedic injury. About 40% of interventional cardiologists had spine problems, consisting of lumbar (70%) or cervical (30%) spine injury. Among physicians with more than 20 years’ experience, spine complaints were found in 60%. About 20% had hip, knee, or ankle injuries. About one-third of respondents missed some work because of injury. These studies did not comment or attempt to quantify how these injuries affected their own quality of life or how injuries might have impaired the physician’s procedural performance.

## 4. Our Method of Work and the Effect of Fatigue on Outcome of Intervention

Occupational hazards aside, the method of how we perform interventions should also be critically reviewed. Percutaneous interventions are performed with the physician standing by the side of the patient, manipulating catheters while looking at the fluoroscopic images on a monitor across the procedure table. For a coronary intervention, the angiogram is obtained and decisions regarding balloon and stent diameter and length are made using our eyes and experience. The stent is positioned based on an image projected some distance from the operator and typically across the procedure table, with the operator sometimes struggling to see clearly. Thus, it is not uncommon for the stent or balloon to miss its mark and it is not uncommon that additional stents are required to cover disease unintentionally missed by the initial stent. A method offering greater precision in both selecting stent length as well as assisting in the ultimate positioning of the stent could improve patient outcomes and reduce costs.

Finally, the effect of operator fatigue on the outcome of a cardiovascular intervention has not been studied. Is the outcome of a multi-vessel intervention performed by a rested interventional cardiologist as a first case at 8 a.m. different if the same interventional cardiologist performed it after six other procedures and at 5 p.m. instead? What if that interventional cardiologist is one of the 50% of individuals with back, neck, or hip pain? Physicians may refuse to publicly acknowledge this limitation, as they believe themselves immune from the effects of fatigue. Again, methods to reduce operator fatigue and increase operator comfort can only lead to improved patient outcomes.

## 5. Rationale and Description of Robotic Intervention

Robotic-assisted intervention holds promise to address many of these issues. In addition to greatly reducing operator radiation exposure, robotic interventions allow the operator to eliminate leaded garments and to sit down during the procedure, thus reducing orthopedic injury and operator fatigue. Further, the fluoroscopic images are in close proximity to the operator and the device allows lesion measurement to enhance precision of the intervention. Currently, the only available device for performance of robotic intervention is the CorPath GRX (Corindus Vascular Robotics, Boston, MA, USA). This is a second-generation device and some of the shortcomings of the first device have been improved upon (CorPath 200).

**Figure 1:**
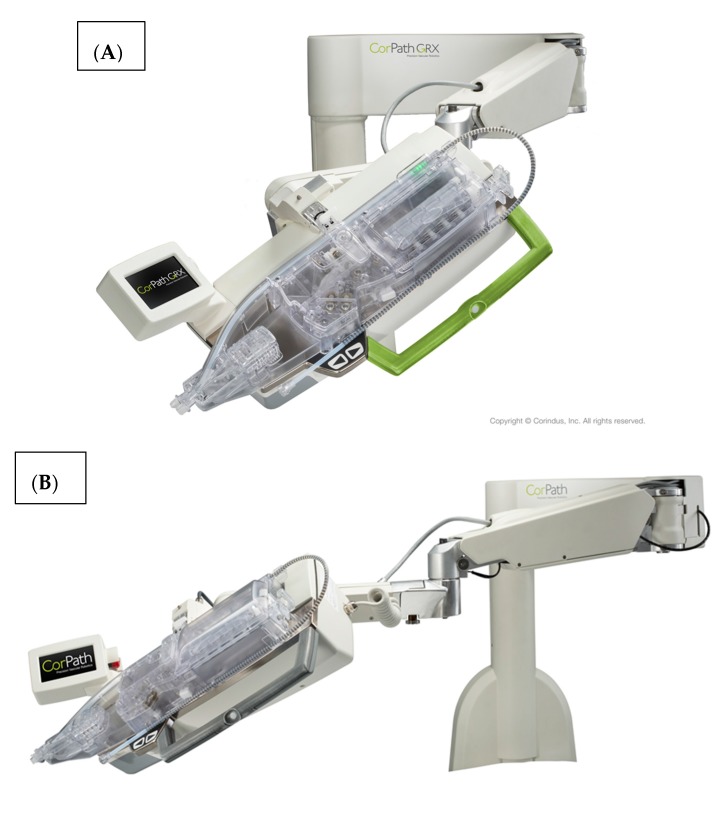

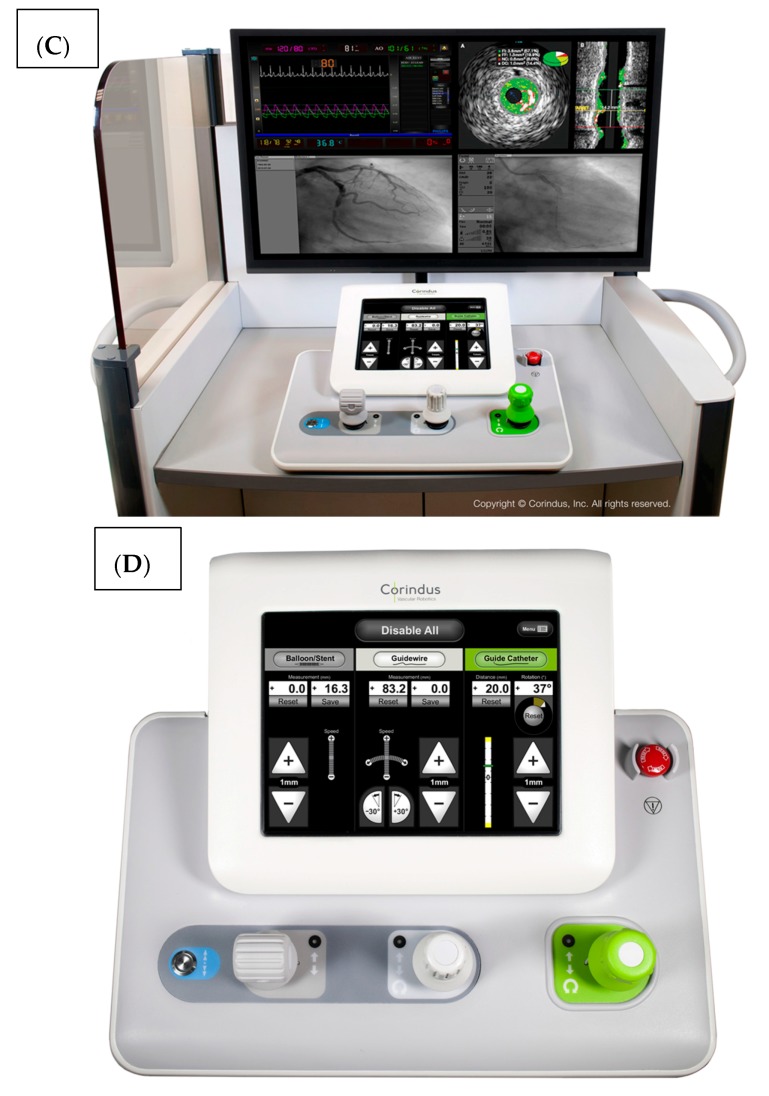
The CorPath GRX system. The robotic arm and disposable cassette is shown in (**A**). The arm extends freely from the table mount to allow variable configurations for catheter attachment (**B**). The interventional cockpit is shown in (**C**) with a close up of the workstation showing the joystick controls in (**D**). (Provided by and with permission from Corindus Vascular Robotics, Boston, MA, USA).

The system is shown in Figure 1. The robotic device (Figure 1A,B) is installed on the procedure table and attaches to the guide catheter. A technician manually loads any desired 0.014” guide wire, rapid exchange balloon, and stent catheter. Meanwhile, the physician sits comfortably (without lead) in a radiation shielded ‘cockpit’ facing a monitor projecting the fluoroscopic images (Figure 1C). The cockpit consists of a touchscreen workstation with three joysticks (Figure 1D). One joystick manipulates the guide catheter allowing it to be gently advanced, retracted, or rotated. Another joystick manipulates the 0.014” guide wire allowing it to be advanced, withdrawn, and torqued with as much freedom and precision as a manually driven guide wire. The third joystick simply advances or retracts the balloon or stent catheter. A measurement tool allows the operator to directly make millimeter measurements of lesion length with either the balloon catheter or guide wire.

In essence, the robot advances and retracts wires and balloon or stent catheters and allows subtle manipulation of the guide catheter. It does not obtain arterial access, exchange guide catheters, or perform angiography. It can advance and retract a pressure wire to perform fractional flow reserve and will allow advancement of intravascular ultrasound for some systems (Phillips Volcano, Phillips Healthcare, Bothell, WA, USA). It does not allow stand-alone performance of an atherectomy device (rotational, orbital, or laser) and can only be used with rapid exchange balloon and stent platforms. It can be used with both radial and femoral access and has been successfully employed to treat complex lesion subsets including multi-vessel coronary disease, left main disease, acute coronary syndromes, vein graft, internal mammary interventions, and some peripheral vascular interventions. FDA approval for endovascular peripheral intervention is anticipated but presently off-label with the second-generation robot.

A growing body of evidence supports the proposed benefits of robotic-assisted PCI. The pivotal trial using the first generation device found that the robotic-assisted procedures were successful in 97.6% of patients with no adverse events attributed to the robot [16]. Importantly, this study confirmed the dramatic reduction in radiation exposure; operator exposure in the cockpit was 95.2% less than if they were at the tableside. Robotic assistance is also associated with less fluoroscopy time, lower contrast dose, and lower radiation exposure to the patient [17]. While it has been difficult to demonstrate improved patient outcomes, it is clear that geographic misses occur commonly with manual methods and are associated with poorer patient outcomes [18]. Robotic-assisted intervention has been shown to be a more precise method of stent delivery with less geographic miss than manual intervention [19,20]. This will likely translate to lower stent costs and better outcomes for patients. The effect of reducing operator fatigue on outcomes is unknown.

## 6. Implementation of a Robotic-assisted Interventional Program: Physician, Institutional, and Educational Considerations

At our institution, we have wholly adopted robotic PCI in an effort to push the boundaries of coronary intervention. The limitations of manual PCI—such as radiation, ergonomics, and safety—are otherwise accepted as ‘costs of business’, but with the advent of robotic PCI we are now able to challenge the notion that these mature techniques are immutable. Now we have an alternative.

The operators and institutions optimally suited to implement a robotic PCI program share features with those who identify as innovators and early adopters of other forefront technologies [21] within cardiovascular medicine such as transradial access, chronic total occlusion, and structural heart and complex peripheral vascular interventions. The advantages of being early adopters include the opportunity to help define the course of progress in the field by providing feedback to innovators and to simultaneously help spread information to others as this technology continues to evolve.

### 6.1. Physician Considerations

The performance of robotic PCI differs from manual PCI in several ways by virtue of removal of the physician from the tableside. While deriving the benefit of improved ergonomics and radiation avoidance, the physician must also surrender the tactile feedback from manual manipulations to which we are so accustomed. Indeed, one of the key skills possessed by an experienced interventionalist is knowing when something ‘feels wrong’ and how to incorporate that feedback into an algorithm of appropriate corrective steps. The robotic interventionalist learns to rely more completely on visual feedback, a scenario similar to the reliance on the visual feedback when coaching an interventional fellow. Additionally, when working from the interventional cockpit, we are physically removed from the patient’s side, where ample verbal and nonverbal clinical information is gleaned by noting the patient’s expressions. This can be easily overcome by the use of closed-circuit cameras that are installed as part of the robotic system, allowing one to continue to feel connected to the patient during treatment. Finally, by working from the cockpit position, we have also placed the assisting operator (fellow, technologist, other) in a de facto primary position, which may create clinical demands that the assistant will need to master, such as catheter exchanges of the terminal portion of monorail equipment. Training on these maneuvers is provided by Corindus and is supplemented by supervising physicians. The closed-circuit camera also allows the operator to observe and monitor these exchanges. Ultimately, robotic PCI is different and creates novel considerations for all members of the interventional team. We believe that the augmented role for the assistant increases staff engagement and is a significant benefit to the system.

### 6.2. Institutional Considerations

The robotic PCI system requires a significant capital outlay as well as a nominal per-case disposable cost structure for the interventional cassette. Similar to other capital and disposable catheterization lab costs, these costs are negotiable with the manufacturer and must not be seen as an obstacle but an investment in the human resource of physician safety, as well as a part of a larger commitment to radiation safety for staff and patients alike. Further, insofar as cases may be able to be performed with additional accuracy and less geographic miss, there is the potential for cost-savings in terms of per-case stent use. Our institution has recently redoubled its commitment to safety, and our acquisition of the robot was made under the auspices of an initiative called ‘Be Safe’, with the goal of making University of Virginia Health System the safest place in the country to give and receive care, both for patients and providers. It would seem obvious that other institutions would want to achieve similar goals.

Robotic PCI creates a cultural shift in the catheterization lab where patient and team safety and clinical accuracy are celebrated. This shift has the potential to create some temporary anxiety as team members learn their new roles, but no significant growth can occur without change. Some health systems have identified robotic medical and surgical care delivery as an opportunity to achieve a competitive advantage within their consumer markers. This works by appealing to patients and referring providers who wish to be at the cutting edge and will choose to seek care at centers with advanced programs. We have not pursued robotic PCI for those particular reasons, but they may have merit in certain circumstances. We see robotic PCI differently, as a treatment modality that may ultimately become the standard of care rather than a marketing ploy.

### 6.3. Educational Considerations

There are three basic ways to approach the educational training of interventional fellows in a robotic PCI program: (1) early incorporation, whereby trainees perform robotic PCI from the beginning of their fellowship; (2) phased-in training, whereby fellows focus on manual techniques for a prescribed duration or case number, then begin to learn robotic manipulation; and (3) late incorporation, whereby fellows are exposed to robotic PCI towards the end of their training as an advanced technique. We advocate early incorporation at UVa, with the idea that trainees can learn manual and robotic PCI (including the assistant’s role) in parallel. This is predicated on the assumption that the program has adequate interventional case volume that would support learning both modalities appropriately.

For seasoned interventionalists and trainees alike, there is a notable and steep learning curve for the application of robotic PCI. This is primarily a function of calibrating one’s hands and mind, and translating joystick maneuvering into mechanical actions, rather than challenges created by the inherent complexity of the system. From a practical standpoint, there is no set number of cases after which an operator is ‘trained’ per se. In our experience, within the first 10 cases, one becomes readily familiar and comfortable with the operation of the system. However, case ‘density’ is more important than case volume (10 cases in two weeks is undoubtedly better than 10 cases in two months). For this reason, we advocate the following protocol:

- For the first five robotic PCI cases, consider choosing cases that are straightforward lesions, such that interventional complexity is not a consideration, with a focus on setup and execution of robotic wiring, catheter manipulation, and interventional catheter delivery. If possible, consider scheduling any elective PCI cases on a single day so as to maximize team experience.
- For the next 5–10 cases, consider selecting increasingly complex cases, so that comfort is gained with more difficult lesions, but without the concomitant need for aggressive guide catheter manipulations.
- As case volume and experience grows, consider focusing on more moderate to difficult cases where maximal radiation savings are possible.

Robotic PCI is ideally suited to simulator training. One of most challenging engineering and design aspects of PCI simulation is the creation of an interventional haptic that closely replicates the tactile feedback experience of a live intervention. With robotic PCI, that element is removed and need not limit the value of the simulated training experience. If incorporated into a formal training program, the opportunity to perform off-line PCI cases holds the promise of dramatically improving performance during clinical interventional cases, thereby reducing risk to patients and allowing learners to master techniques at their own pace. In the future, we believe that robotic PCI will provide a fertile ground to experience virtual cases at varying levels of complexity prior to, or in concert with, clinical cases during training.

Once the learning curve is overcome, it has been our experience that the total procedure time for a robotically assisted procedure is not significantly different than a manually performed procedure, however, it is important for the staff and facility to understand that procedure times will be longer during the initial implementation phase while overcoming the learning curves. The more cases are performed robotically, the faster the setup is performed and the shorter the delay.

## 7. Ideal Cases for Robotic PCI

Case selection is key in robotic PCI, much as it is with any adjunctive technology such as atherectomy or hemodynamic support cases. The ideal case description evolves over time as operators become increasingly accomplished with the techniques involved. Nonetheless, one must consider the patient–lesion complex when considering the ideal cases for robotic PCI. Even a straightforward lesion in an unstable or high-risk patient may not be an advisable target, whereas a more difficult lesion in a very stable patient could be an excellent opportunity for robotic assistance.

**Figure 2:**
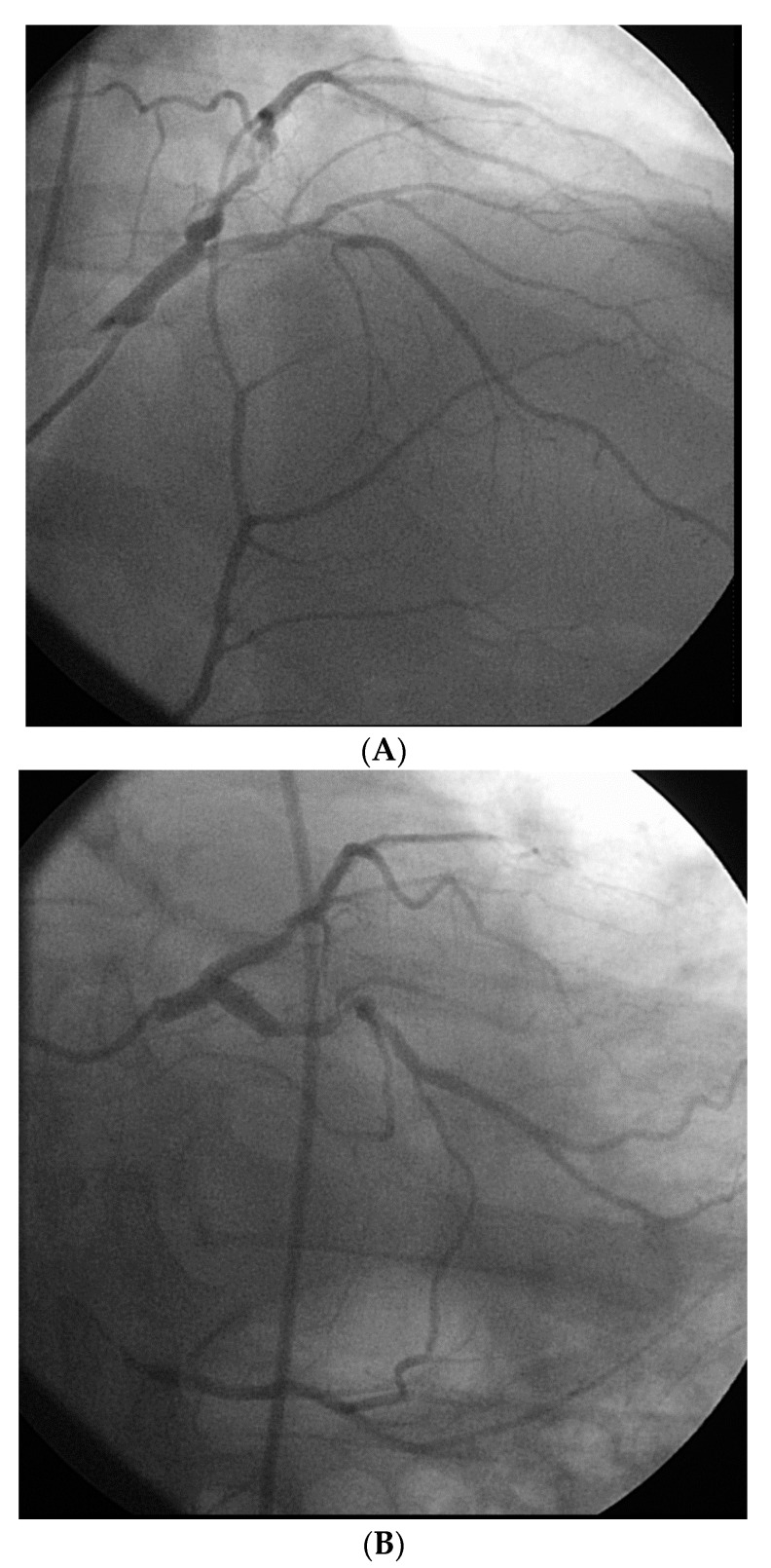

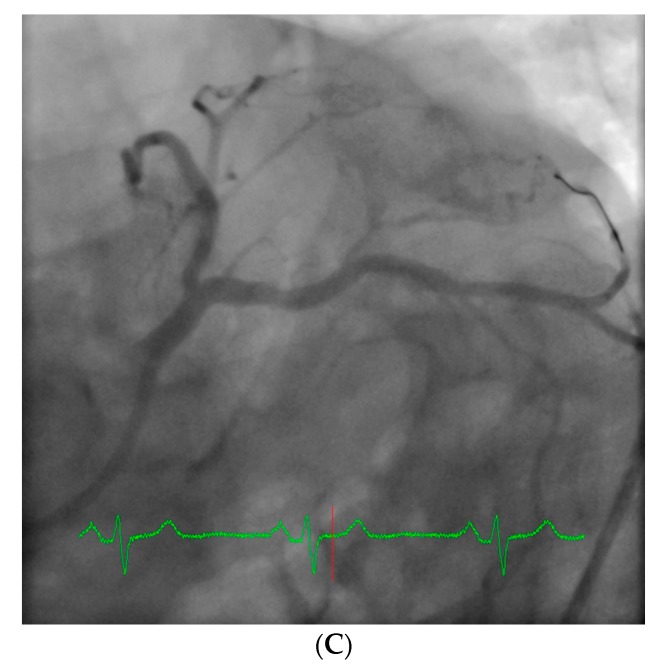
Example of an ideal case for robotic PCI. These images are from a 72 year-old with unstable angina referred to our hospital for intervention. Diagnostic catheterization showed moderate disease in the LAD (**A**) but with fractional flow reserve of 0.65 and severe disease of the LCX (**B**) with chronic occlusion of the RCA. The LAD and LCX were both treated successfully robotically with post-stent images shown in (**C**).

However, an ideal robotic intervention is one where the operator obtains access and performs initial guide placement manually, and is then able to complete the entire intervention from the cockpit without having to re-scrub, even briefly, to perform some portion manually. An example of an excellent case for robotic PCI is a two vessel PCI of the left anterior descending and circumflex arteries, where one guide shape and a single guidewire is used for both vessels, and where significant radiation exposure or a prolonged time in lead are both avoided. Figure 2 shows an example of an ideal robotic PCI case.

## 8. Poor Choices for Robotic PCI

Patients with clinically unstable presentations may not be ideal candidates for robotic PCI, particularly early in the operator’s experience. Cases such as acute ST-segment elevation myocardial infarction, PCI of a single remaining coronary, or PCI in patients with profound depression in ejection fraction or with severe clinical heart failure may decompensate rapidly and thus, being physically present at the bedside may be preferable. Interestingly, patients with left ventricular support devices in place may be very reasonable to treat robotically because of the clinical stability afforded by these devices.

**Figure 3:**
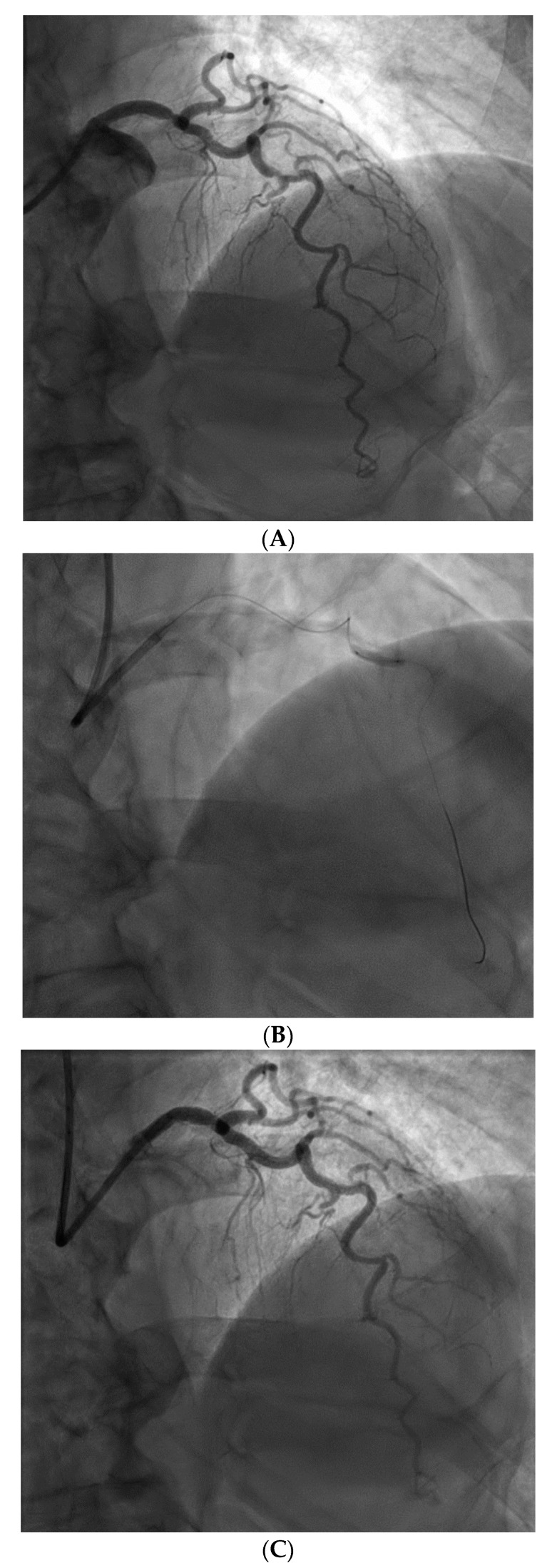
Example of a complex lesion involving the LAD in a patient with angina and severe tortuosity (**A**). Despite the tortuosity, the lesion was successfully wired, ballooned, and stented robotically (**B**). Post stent result shown in (**C**).

**Figure 4:**
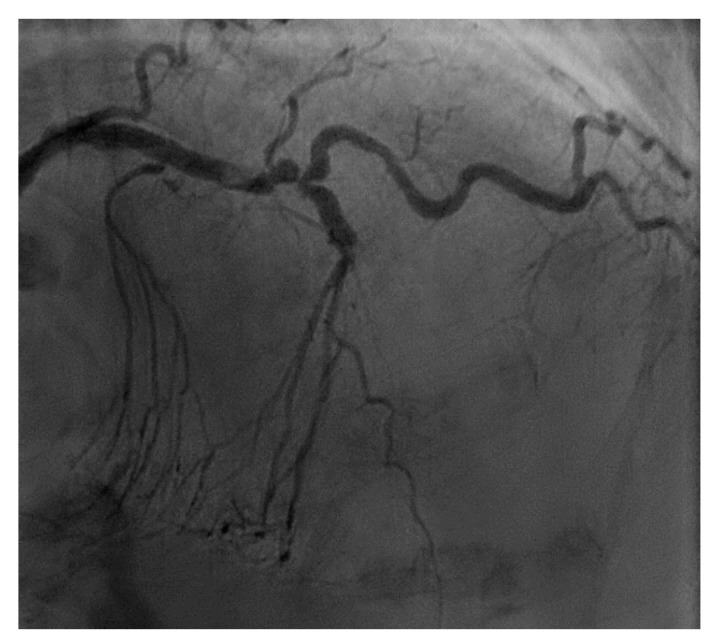
This is an example of a poor choice for robotic PCI. The patient has a single coronary artery with an ejection fraction of 20% and the lesion is very complex in the artery with severe tortuosity and angulation.

**Figure 5:**
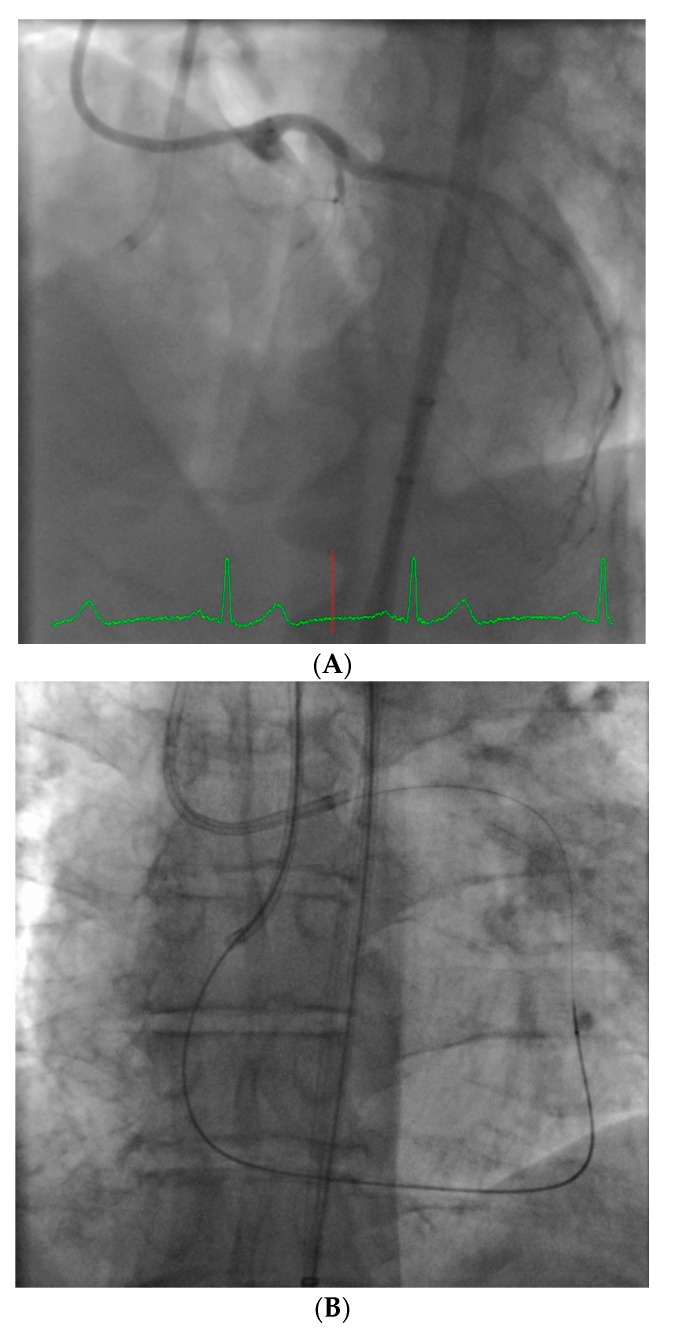

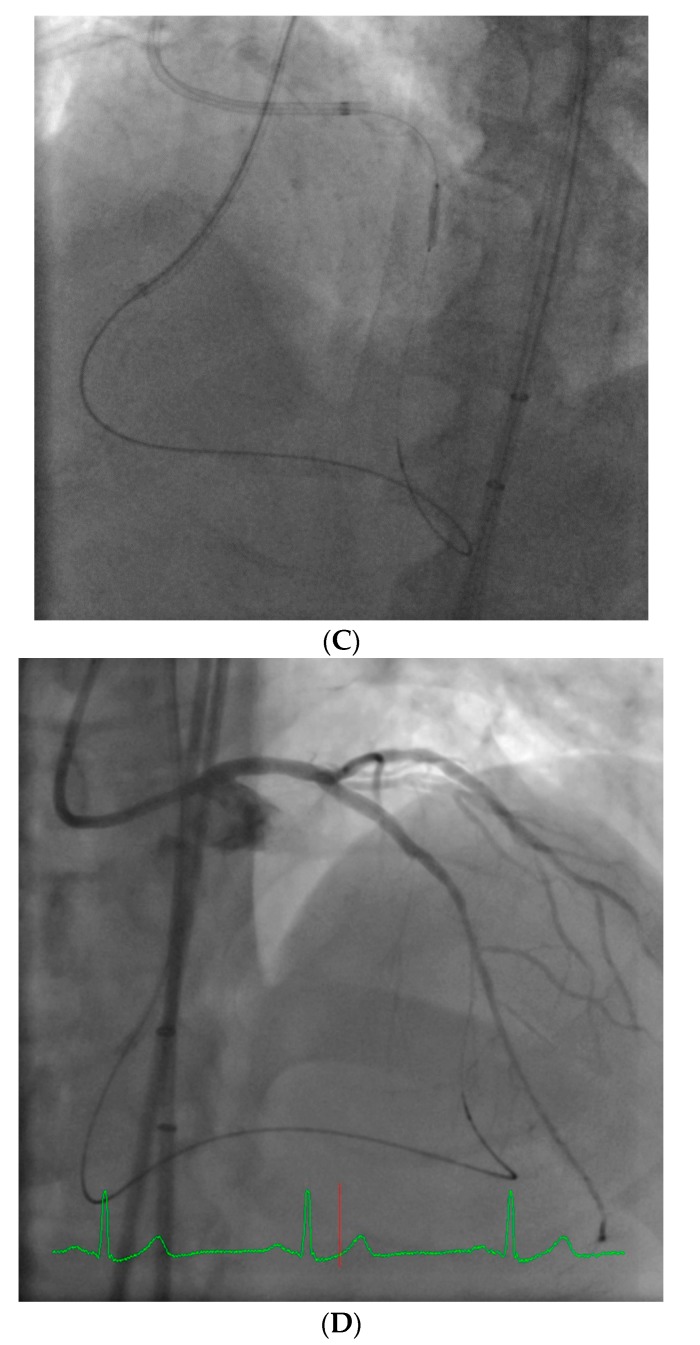
These images are from a 52 year-old with angina and a chronic total occlusion of the LAD (**A**). The lesion was crossed successfully using retrograde techniques from the RCA; this step was performed manually (**B**). After the retrograde wire was externalized, the procedure was converted to robotic assistance and subsequent balloon, stenting, and post-stent balloon dilatations were performed with the operator in the cockpit, thus reducing radiation exposure by 50% (**C**). The final result is shown in (**D**).

**Figure 6:**
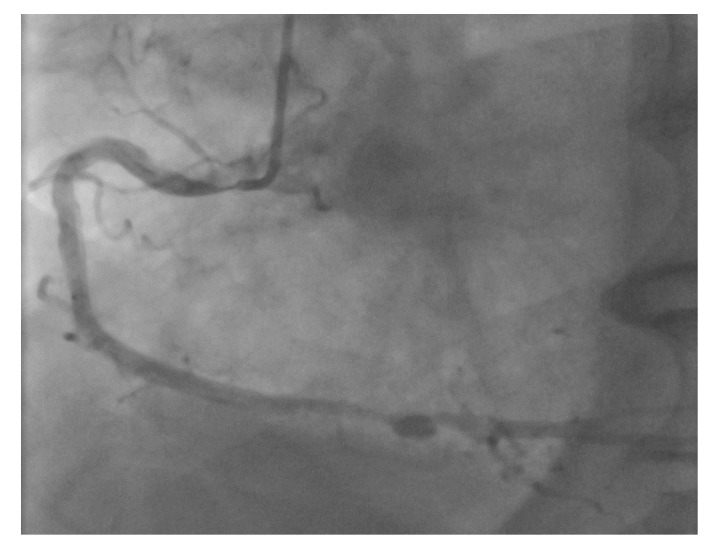
Ostial RCA lesions can be very challenging to perform robotically due to the complex interactions between the guide catheter position and stent catheter.

From a pure anatomical standpoint, bifurcation cases with planned kissing stents or kissing balloon angioplasty are not ideal for robotic PCI primarily because the system can only advance one catheter actively. However, bifurcations where provisional side-branch intervention is planned are reasonable choices for robotic PCI. Cases with excessive vessel tortuosity may be successfully performed robotically (Figure 3); however, extreme arterial angulation, or lesions that are severely eccentric or have dissected plaque morphology may require the use of tactile feedback that is not afforded by the robot and are not ideal (Figure 4). Although the advanced wire techniques required for anterograde wire escalation, dissection re-entry approaches, or retrograde approaches in chronic total occlusion intervention are not applicable robotically, we have used the robot to deliver and perform balloon angioplasty, stenting, and post-dilatation after first successfully manually crossing chronic total occlusions with a guidewire (Figure 5). Although the current generation system allows the operator to make fine adjustments in the guide catheter position, ostial lesions, particularly involving the RCA can be very challenging to perform robotically (Figure 6). Finally, cases that require the use of aspiration, atherectomy, filter wires, or certain imaging catheters (OCT and some IVUS), are not suitable for stand-alone robotic PCI.

However, it is worth noting that robotic PCI should not be considered ‘all or none’. Rather than defining what percent of cases are “robot cases”, we think it is more appropriate to consider the question: “What portion of any specific intervention can be done robotically?” Particularly in long and complex cases, if a significant amount of the procedure can be done robotically, it is worthwhile to include the robot in the case in order to limit operator radiation exposure. Therefore, we frequently perform ‘hybrid’ interventions where manual manipulations are done for some steps (such as wire crossing in a chronic total occlusion intervention, or lesion preparation with atherectomy devices) and the robot is used for the routine balloon and stent portions.

## 9. Obstacles to Implementation and Future Directions of Robotics in the Cardiac Catheterization Laboratory

Frustrations with early adoption of a new technology are easier to accept when there are no other choices (for example, the first generation transcatheter aortic valve replacements and dial up internet access). However, one of the challenges of wide-spread adoption of robotic PCI is the fact that manual techniques are already well-refined procedures and operators are extremely comfortable with wire and catheter manipulation. Unlike other new technology that offers the operator access to a novel technique or procedure, the robot simply changes the way we already perform an existing procedure. Thus, implementation of robotic PCI will inevitably slow down the operator, especially in the early phases. Operators may become frustrated with any delay since they know they can quickly perform a step manually that initially may take them a bit longer with the robot. A successful program requires the operator and staff to commit to the value of reducing operator fatigue, injury, and radiation exposure over the long term. Again, it is a shift in the mindset in changing the way we work.

The second-generation device represented a significant improvement in the performance of robotic PCI with the addition of guide catheter control, allowing the operator to push the guide catheter forward if it backs out during balloon or stent catheter advancement, make minor adjustments in coaxial positioning if there is difficulty advancing catheters, and gently back out the guide catheter in the event of an ostial lesion. This greatly enhanced our ability to perform robotic PCI on lesions we might otherwise have chosen to perform manually. In fact, we have successfully reengaged both right and left guide catheters that completely disengaged from the coronary artery by using the guide controls.

Robotic PCI technology will continue to improve with future generation devices. It would be ideal if engineers could add additional active ports for advancing catheters to allow easier methods to treat complex bifurcation lesions, which are currently very tedious to perform robotically. Further enhancements would allow the use of over the wire techniques, peripheral applications, and manipulation of other coronary devices. However, it is unlikely that robotic techniques will be applicable to structural interventions. We are hopeful that more widespread adoption of this novel way of working in the cardiac catheterization laboratory will lead to further advances, thus making PCI procedures safer and more efficient for both the patient and the operator.

## Figures and Tables

**Table 1:** Occupational Hazards of Interventional Cardiology.

Hazards of Radiation Exposure	Hazards of Protection from Radiation Exposure	Other Hazards
1. Cancer Basal cell skin cancer Chronic myelogenous leukemia Thyroid cancer Brain tumor	1. Orthopedic Injury Lumbosacral spine Cervical spine Hip Knee Ankle	Exposure to blood-borne infections
2. Cataracts	2. Operator Fatigue	
3. Effects on reproductive health Low sperm count Teratogenesis		
4. Accelerated atherosclerosis		
5. Neurodegenerative changes		
